# Changes in the Foveal Outer Nuclear Layer of Central Serous Chorioretinopathy Patients Over the Disease Course and Their Response to Photodynamic Therapy

**DOI:** 10.3389/fmed.2021.824239

**Published:** 2022-01-14

**Authors:** Kaixin Deng, Yufei Gui, Yi Cai, Zhiqiao Liang, Xuan Shi, Yaoyao Sun, Mingwei Zhao

**Affiliations:** ^1^Department of Ophthalmology, Peking University People's Hospital, Beijing, China; ^2^Eye Diseases and Optometry Institute, Beijing, China; ^3^Beijing Key Laboratory of Diagnosis and Therapy of Retinal and Choroid Diseases, Beijing, China; ^4^College of Optometry, Peking University Health Science Center, Beijing, China

**Keywords:** central serous chorioretinopathy, outer nuclear layer, photodynamic therapy, disease duration, OCT

## Abstract

**Objective:**

To investigate the association between foveal outer nuclear layer (ONL) thickness and the natural course of central serous chorioretinopathy (CSC), as well as the thickness change after photodynamic therapy (PDT), exploring the PDT timing for CSC.

**Methods:**

This retrospective consecutive case series included 358 CSC patients between January 2014 and December 2019. All patients were divided into four groups depending on disease duration: Group A: ≤1 month; Group B: >1 and ≤3 months; Group C: >3 and≤6 months and Group D: >6 months. Foveal ONL thickness of the CSC eye and the clinically healthy fellow eye were measured and compared in all patients. Fifty-six patients were successfully treated with half-dose of PDT, showing complete subretinal fluid absorption, were followed up for more than 6 months and further investigated. The recovery of foveal ONL thickness was analyzed in the affected eyes of patients with different disease duration.

**Results:**

No significant reduction was found in CSC foveal ONL thickness (μm) compared to the fellow eye in patients with disease duration less than 1 week (112.3 ± 12.2 vs. 116.7 ± 15.3, *P* = 0.268). Patients with longer disease duration had varying degrees of ONL thinning compared to the contralateral eye (all *P* < 0.05) and this difference was more pronounced in patients with disease duration greater than 6 months (75.8 ± 12.9 vs. 113.0 ± 11.5, *P* < 0.001). At 6-month follow-up after PDT, foveal ONL thickness of patients with <1 month disease duration recovered significantly from onset (97.3 ± 18.2 to 113.6 ± 8.7, *P* < 0.001) and became similar to that of the healthy fellow eye. Foveal ONL thickness of patients with duration>1 and≤3 months recovered significantly (88.5 ± 11.5 to 95.8 ± 11.3, *P* = 0.012) but remained thinner than that of the healthy fellow eye. Foveal ONL thickness did not improve significantly in cases with disease duration longer than 3 months (*P* > 0.05).

**Conclusion:**

Foveal ONL thinning was positively associated with disease duration prior to treatment suggesting that longer disease duration limits scope for foveal ONL recovery. CSC patients should be treated with PDT as soon as possible to prevent disease development and reduced visual function.

## Introduction

Albert V Graefe reported central serous chorioretinopathy (CSC) in 1866, describing serous detachment of the macula and serous pigment epithelial detachment (1, 2). The condition primarily affects emotionally stressed men aged 20 to 45 years old (2, 3) and is a severe hazard to vision in middle-aged men. Visual distortion, micropsia, and impaired vision may be their main complaint (3). Increased glucocorticoid levels, A-type personality, hypertension, peptic ulcer, *Helicobacter pylori* infection, and sleep apnea syndrome are all believed to be related to CSC (4–6). Acute CSC is thought to be self-limiting to some extent, with neurosensory detachment healing without intervention in months (4, 7). However, in almost half of CSC cases, healing does not occur unaided, so effective therapy is critical (8). Photodynamic therapy (PDT), risk factor intervention, micro-pulse laser therapy, choroidal salt corticoid receptor antagonist, and even anti- vascular endothelial growth factor (VEGF) medication therapy are some of the current treatment options (9–12). In terms of subretinal fluid absorption rate and final visual acuity improvement, PDT is considered superior to other therapies (13).

CSC is classified into three forms based on the clinical presentation and course of the disease: acute CSC, chronic CSC, and bullous retinal detachment. Acute CSC is characterized by a period of symptoms and/or retinal detachment, as well as monofocal or multifocal retinal pigment epithelium leakage on fundus fluorescein angiography (FFA). Chronic CSC may present with multifocal or diffuse retinal pigment epithelium depigmentation with a serous retinal detachment and can be followed by neovascularization (14). Microstructural changes in optical coherence tomography (OCT) of CSC patients are closely associated with the disease courses. For example, Ozgur et al. found that photoreceptor cell layer atrophy occurs around 4 months after the onset of symptoms (15). Ozdemir et al. discovered that photoreceptor loss could start within the first 3 months of CSC, and that the thickness of outer nuclear layer (ONL, photoreceptor as primary structure) was negatively correlated with the duration of symptoms (16). Although macular microstructural changes have been linked to the course of CSC, other microstructural changes in the macula of CSC patients in relation to the disease course have been studied less thoroughly.

The retinal outer nuclear layer (ONL) comprises the nuclei of the photoreceptor cells (12) and is the most essential component in the central macular fovea. According to recent studies, CSC belongs to the patchy choroidal hypertrophy spectrum of disease, while the thickness of the fovea ONL is decreased (17–19) perhaps due to photoreceptor cell death (16, 20, 21). Matsumoto et al. confirmed that ONL thickness was inversely connected with best corrected visual acuity (BCVA, logMAR) and that the ONL was a robust predictor of visual prognosis (19). According to Masayuki Hata, ONL thinning continues while subretinal fluid (SRF) is present (21). Despite the above studies, no systematic studies have linked changes in the ONL thickness to the course of CSC disease.

In CSC patients, changes in the ONL are also linked to recovery. CSC patients usually recover well after undergoing PDT. Previous studies have shown a gradual increase in ONL thickness after CSC remission, with simultaneous improvement in visual acuity. This is consistent with suggestions that the ONL can be repaired and visual function restored (19, 22). Moreover, despite ONL thickness being significantly linked to CSC, no systematic studies have investigated the ONL response to PDT in patients with different disease courses. In addition, although PDT has been shown to be safe and effective in the treatment of CSC, some ophthalmologists still believe that treatment may be delayed while CSC patients can be observed first (4). As a result, the timing of PDT treatment remains controversial. An understanding of the correlation between ONL recovery and PDT timing will provide evidence for the latter in CSC treatment.

To improve understanding of the above issues we conducted this retrospective consecutive case series reviewing foveal ONL changes of CSC disease duration in 358 patients. Fifty-six patients in whom, treated successfully with half-dose of PDT, showing complete subretinal fluid absorption and followed up for more than 6 months, were further investigated. The link between foveal ONL thickness recovery disease duration was investigated.

## Materials and Methods

We retrospectively analyzed 358 patients diagnosed with CSC between January 2014 and December 2019 in the department of ophthalmology, Peking University People's Hospital. The diagnosis was made using multimode imaging [spectral domain OCT (SD-OCT), FFA and indocyanine green angiography (ICGA)]. Patients with primary, monocular onset CSC, their fellow eye unaffected, were eligible to participate. Patients with other primary retinal disorders, such as epi-retinal membrane, rhegmatogenous retinal detachment, uveitis, age-related macular degeneration, polypoid choroidal vasculopathy, high myopia fundus changes and tumors, were excluded. Patients with a history of PDT or other laser photocoagulation, as well as anti-VEGF treatment, were also excluded. Patients who were treated with half-dose PDT and had complete subretinal fluid absorption with follow up for more than 6 months were analyzed as a subgroup.

**Figure 1:**
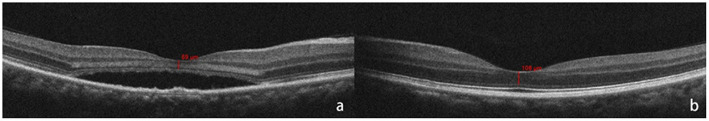
Outer nuclear layer (ONL) thickness was calculated as the distance between internal limiting membrane (ILM), and external limiting membrane (ELM) at the fovea. The red bar shows the measurement of foveal ONL thickness (defined as the distance from ILM to ELM). Optical coherence tomography (OCT) shows SRF at fovea and thinner ONL **(a)** than that of the fellow healthy eye **(b)** at the time of the initial consultation.

Each patient underwent a thorough eye examination and medical history inquiry, including details of the period since symptom onset (duration), best-corrected visual acuity (BCVA), anterior segment examination with slit lamp, posterior segment examination and SD-OCT (Carl Zeiss Meditec, Inc., Dublin, CA), FFA (Carl Zeiss Meditec, Inc., Dublin, CA), and ICGA (Heidelberg Engineering, Heidelberg, Germany). At least two retinal experts had assessed and measured foveal ONL thickness both horizontally and vertically and the average of their results was recorded as fovea ONL thickness. On SD-OCT of fovea, the inner line was defined as the internal boundary of the internal limiting membrane (ILM), and the outer line was defined as the external boundary of the external limiting membrane (ELM). The distance between ILM and ELM at the fovea was used to calculate the foveal ONL thickness (23) (Figure 1). The measurements were completed using the OCT software's measuring tool. Snellen BCVA was converted to logMAR for statistical purposes.

Patients received PDT which was administered using 50% dose of verteporfin according to previously stated (24–26) (Visudyne, Novartis AG, Bulach, Switzerland). Infusion of verteporfin was performed for >10 min, followed by laser delivery at 15 min from the start of infusion. A total light energy of 50 J/cm^2^ was delivered for 83 seconds to the area of choroidal hyperperfusion, as observed on ICGA.

The data were statistically analyzed using the SPSS statistics software version 24.0 (SPSS Inc, Chicago, IL, USA). In order to compare the changes of ONL between different courses of disease, the ONL thickness of the affected eye and the fellow eye were compared using a paired *t*-test in each group. Similarly, in order to compare the ONL changes of the same patients after PDT treatment, the ONL thickness change was also compared using paired *t*-tests for pre- and after PDT. We also analyzed whether factors other than disease duration influenced the change in ONL after PDT treatment. For the categorical variable, a homogeneity of variance was first tested. If there is a homogeneity of variance, the *t*-test was chosen, or else the rank sum test was performed. For continuous variables, a spearman correlation analysis was performed. The statistical significance level was set at *P* < 0.05.

The study was designed in accordance with the Declaration of Helsinki, and the protocol was approved by Peking University People's Hospital's Clinical Research Ethics Committee.

## Results

This study enrolled 358 patients, 251 of whom were men (70.1%) and 107 were women (29.9%) with a mean age overall of 43.5 ± 10.1years.For analysis, patients were categorized into four groups according to their duration (period since onset of symptoms): Group A (duration ≤1 month); Group B (duration>1 and ≤3 months); Group C (>3 and ≤6 months); Group D (duration >6 months). The patients' demographic information is displayed in Table 1.

**Table 1:** Demographic information of patients in groups A, B, C and D.

	**No. of eyes**	**Sex (F/M)**	**Age, mean ±SD (year)**
Group A	91	27/64	41.1 ± 11.9
Group B	92	26/66	43.6 ± 9.1
Group C	67	20/47	42.6 ± 10.0
Group D	108	34/74	47.8 ± 8.4
Total	358	107/251	43.5 ± 10.1

*Group A, duration ≤1 month; Group B, 1<duration ≤3 months; Group C, 3 < duration ≤6 months; Group D, duration >6 months; F, female; M, male; SD, standard deviation*.

### Changes in Foveal ONL Thickness With the Progression of CSC

**Figure 2:**
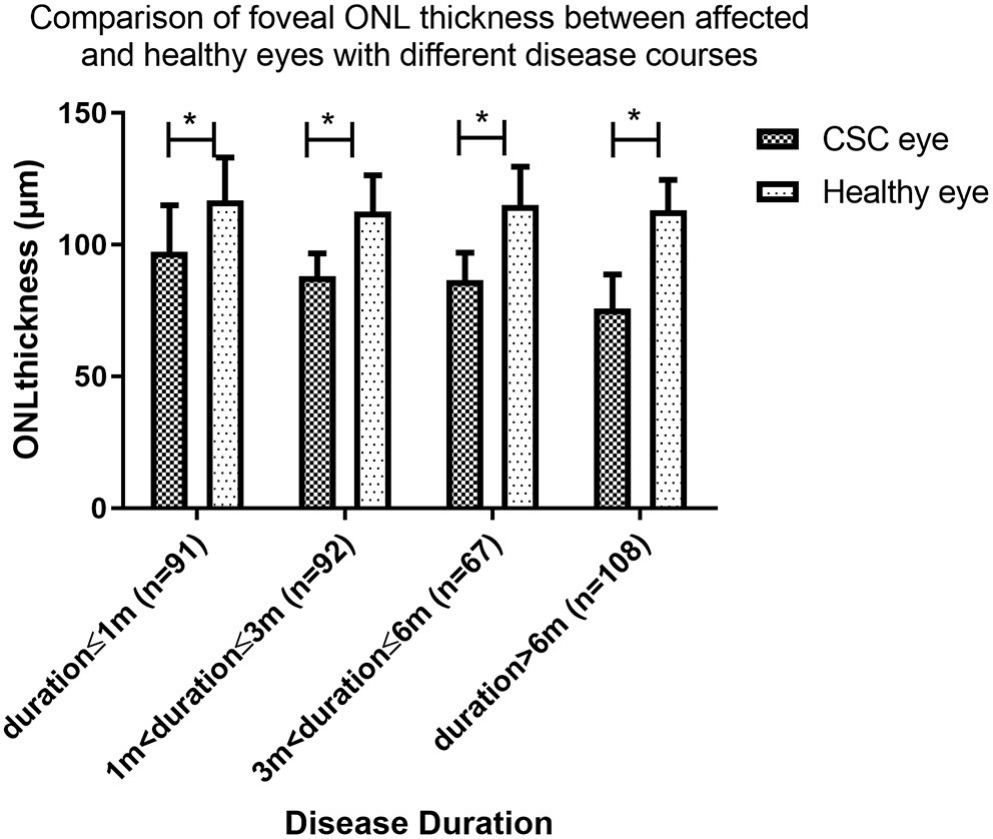
Comparison of foveal outer nuclear layer (ONL) thickness between affected and healthy eyes with different disease courses. The foveal ONL thickness of the affected eye is significantly thinner in all groups (paired *t*-test, *P* = 0.004, *P* < 0.001, *P* < 0.001, *P* < 0.001). (* indicates statistical significance). CSC, central serous chorioretinopathy.

At baseline, OCT measures showed mean foveal ONL thickness of the affected eye were 97.2 ± 17.7 μm, 88.0 ± 8.7 μm, 86.6 ± 10.3 μm, and 75.8 ± 12.9 μm in Groups A, B, C, and D respectively. In all four groups, the foveal ONL thickness of the clinically healthy follow eye was 116.8 ± 16.3 μm, 112.6 ± 13.7 μm, 115.1 ± 14.5 μm, and 113.0 ± 11.5 μm, respectively. In each of these groups the ONL was significantly thinner in the affected eye than the fellow eye (paired t test, *P* = 0.004, *P* < 0.001, *P* < 0.001 and *P* < 0.001, respectively, Figure 2).

**Figure 3:**
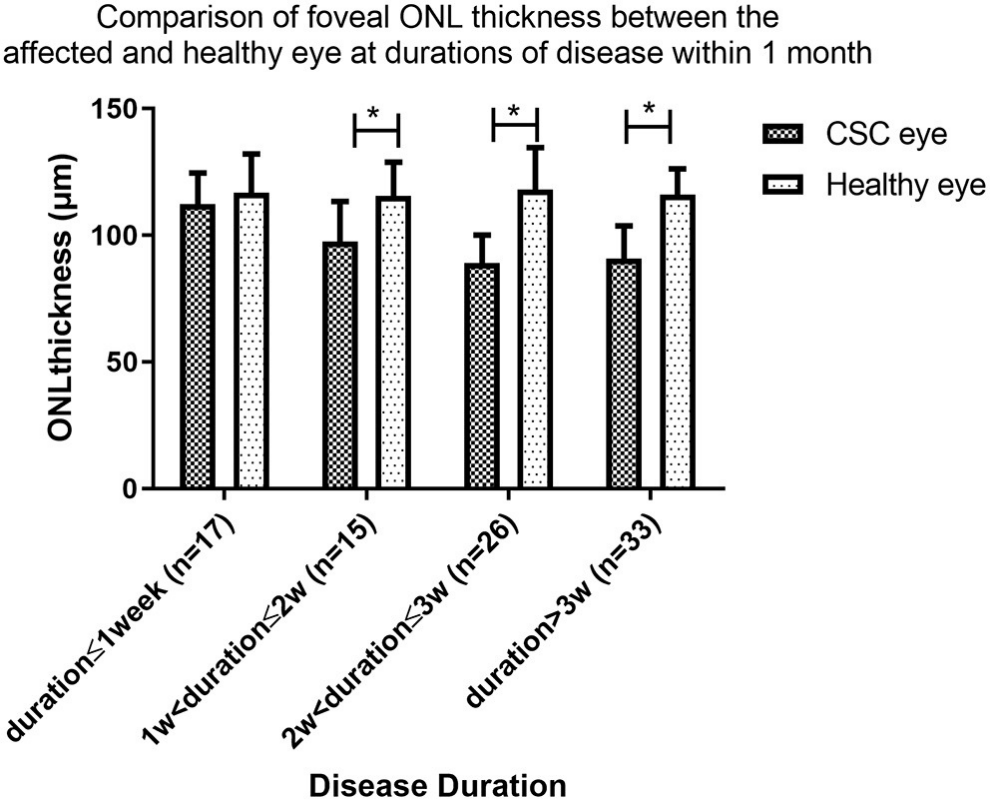
Comparison of foveal ONL thickness between the affected and healthy eye at durations of disease within 1 month. In a subgroup analysis of Group A, ONL thickness was similar in the affected and fellow eyes of patients with central serous chorioretinopathy (CSC) disease duration of ≤1week (*P* = 0.286), but was significantly thinner in the affected than fellow eye in patients in the following groups: (14 weeks; (*P* = 0.009, *P* < 0.001, and *P* < 0.001 respectively). (* indicates statistical significance).

The patients in group A were also subdivided and investigated further. Patients with a disease duration ≤1week had no significant thinning of the ONL thickness compared to the healthy eye (112.3 ± 12.2 μm vs. 116.7 ± 15.3 μm, *P* = 0.286), but significant ONL thinning compared to the contralateral eye was found in patients in the following groups: duration>1 and ≤2 weeks (97.5 ± 15.8 μm vs. 115.6 ± 13.2 μm, *P* = 0.009), duration>2 and ≤3 weeks (88.9 ± 11.1 μm vs. 118.1 ± 16.5 μm, *P* < 0.001), and duration >3 weeks and ≤4 weeks (90.8 ± 12.9 μm vs. 116.0 ± 10.2 μm, *P* < 0.001) (Figure 3).

### Changes in Foveal ONL Thickness After PDT

**Figure 4:**
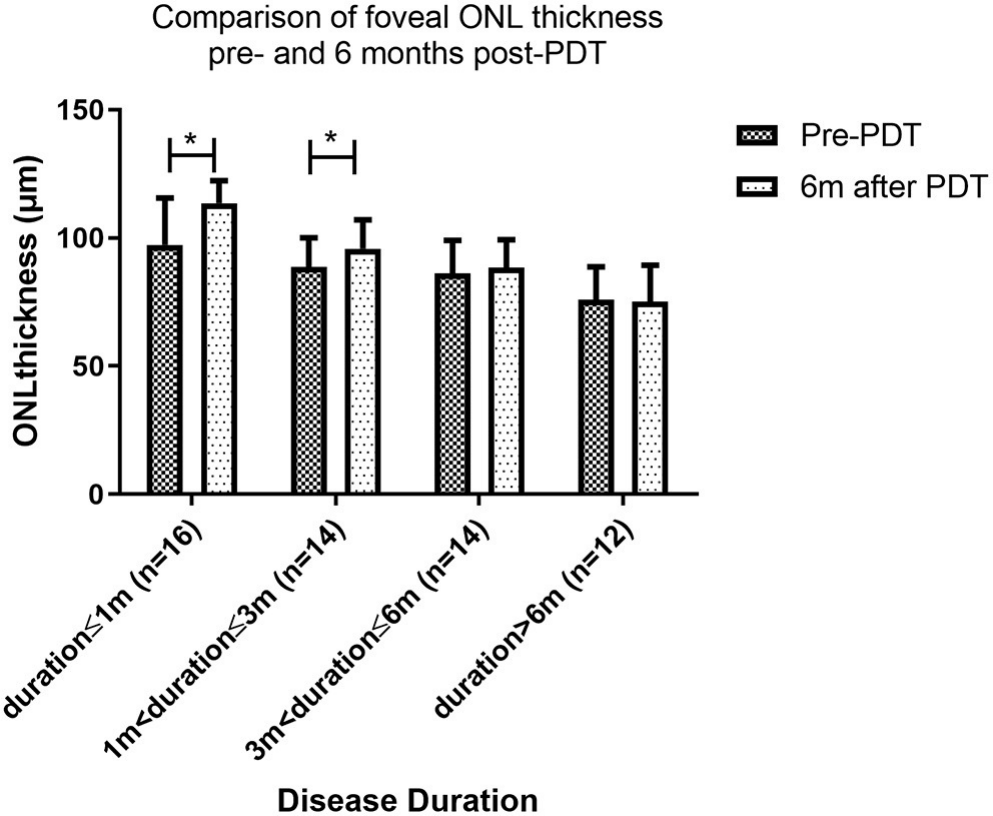
Comparison of foveal ONL thickness pre- and 6 months post-PDT. Patients with a disease duration of less than 1 month had the best ONL recovery, followed by those with a disease duration of 1 to 3 months. Even if the subretinal fluid was totally absorbed, ONL could not be retrieved in patients with a disease duration of more than 3 months (* indicates statistical significance).

To learn more about how foveal ONL changed after patients were treated with PDT, we chose 56 patients who had complete subretinal fluid absorption with 6-month follow-up after PDT. We compared the thickness of ONL pre- and 6 months post-PDT, if there is a statistically significant difference, a significant recovery of ONL was defined. We discovered that the recovery of the ONL after PDT was dependent on the duration of the disease. At 6 months follow up after PDT, significant thickness improvement after PDT was found in patients with duration ≤1 month (97.3 ± 18.2 μm vs. 113.6 ± 8.7 μm, *P* < 0.001) and duration >1 and ≤3 months (88.5 ± 11.5 μm vs. 95.8 ± 11.3 μm, *P* = 0.012). However, no significant change in ONL was found in patients with duration >3 and ≤6 months (86.2 ± 12.9 μm vs. 88.4 ± 10.9 μm, *P* = 0.291) and duration >6 months (76.1 ± 13.2 μm vs. 75.2 ± 14.1 μm, *P* = 0.738). Patients with a disease duration of less than 1 month had the best fovea ONL recovery, followed by those with a disease duration of 1 to 3 months. Even if the subretinal fluid was totally absorbed, ONL could not be retrieved in patients with a disease duration of more than 3 months (Figure 4).

It is worth noting that, after 6 months of PDT, the ONL in patients with duration ≤1 month eventually recovered to a level comparable to that of the contralateral healthy eye (113.6 ± 8.7 μm vs. 116.8 ± 16.3 μm, *P* = 0.380), while the ONL of patients duration>1 and ≤3 months remained thinner than that of the contralateral healthy eye (95.8 ± 11.3 μm vs. 112.6 ± 13.7 μm, *P* < 0.001), despite the significantly recovery from the pre-treatment level (88.5 ± 11.5 vs. 95.8 ± 11.3, *P* = 0.012).

**Figure 5:**
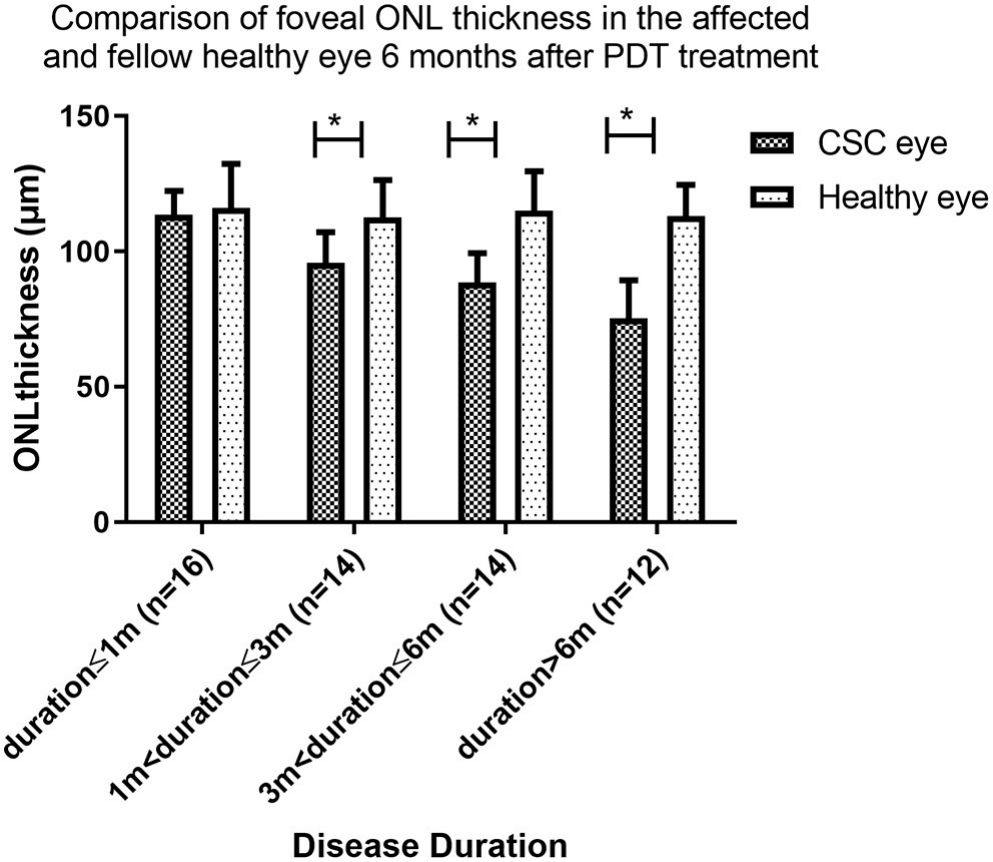
Comparison of foveal ONL thickness in the affected and fellow healthy eye 6 months after PDT treatment. At 6 months follow up after PDT, the foveal ONL thickness in patients with disease duration ≤1 month eventually recovered to a level comparable to that of the contralateral healthy eye. In patients with disease duration over 1 month, ONL was not restored to this level (* indicates statistical significance).

For patients whose duration>3 and ≤6 months and duration >6 months, the foveal ONL showed no recovery compared to the pre-treatment and remained significantly thinner than their fellow healthy eye (88.4 ± 10.9 vs. 115.1 ± 14.5, *P* < 0.001) and (75.2 ± 14.1 vs. 113.0 ± 11.5, *P* < 0.001) (Figure 5).

We also analyzed other factors that may affect the ONL thickness change according to previous references (22, 27), include sex, age, height of retinal detachment, width of subretinal space, ratio of width and height, and BCVA. None of those factors had an effect on ONL thickness changes (Supplementary Table S1).

### Changes in BCVA After PDT

**Figure 6:**
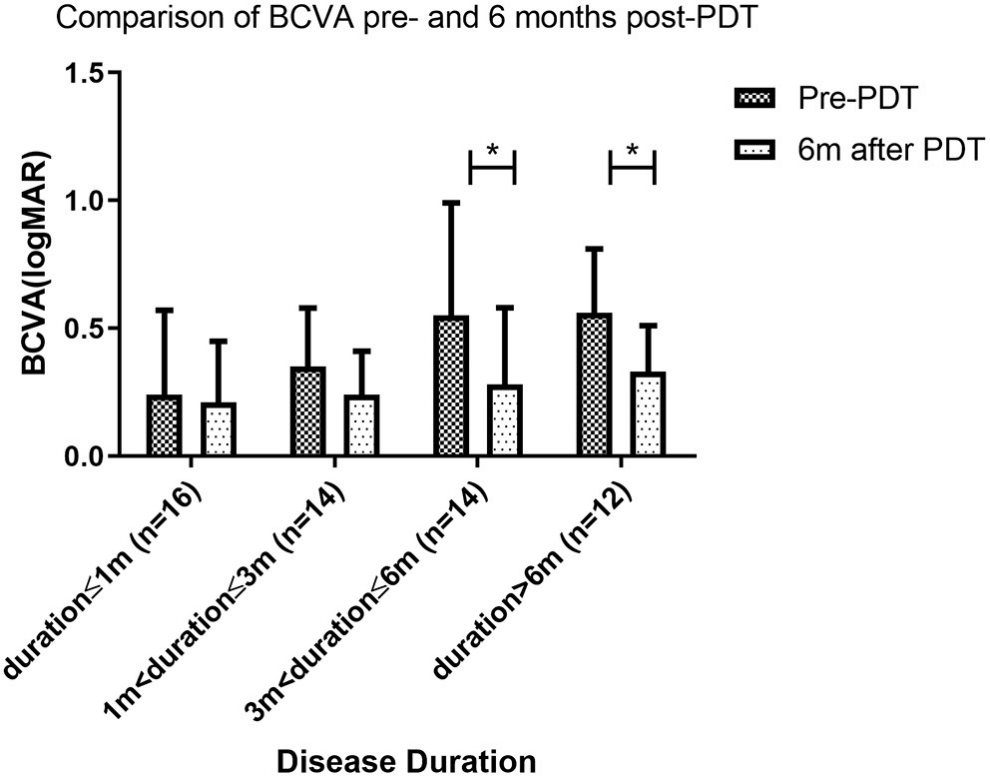
Comparison of BCVA pre- and 6 months post-PDT. At 6 months follow up after photodynamic therapy (PDT), the best corrected visual acuity (BCVA) of patients with disease duration longer than 3 months was significantly better than BCVA pre-PDT. However, BCVA was similar pre- and post-PDT for patients with disease duration of 3 months or less (* indicates statistical significance).

All patients had some degree of visual acuity recovery after PDT. BCVA (logMAR) for patients with duration <1 month, duration>1 and ≤3 months, duration>3 and ≤6 months and duration >6 months was 0.24, 0.35, 0.55, and 0.56 respectively at baseline (before PDT). Visual acuity improved to 0.21 (*P* = 0.573), 0.24 (*P* = 0.136), 0.28 (*P* = 0.0003), and 0.33 (*P* = 0.002) respectively at the 6-month follow-up after PDT. Visual acuity recovery was not significant at disease durations 3 months or lower, and was significant at durations longer than 3 months (Figure 6).

### Typical Cases

#### Case 1

**Figure 7:**
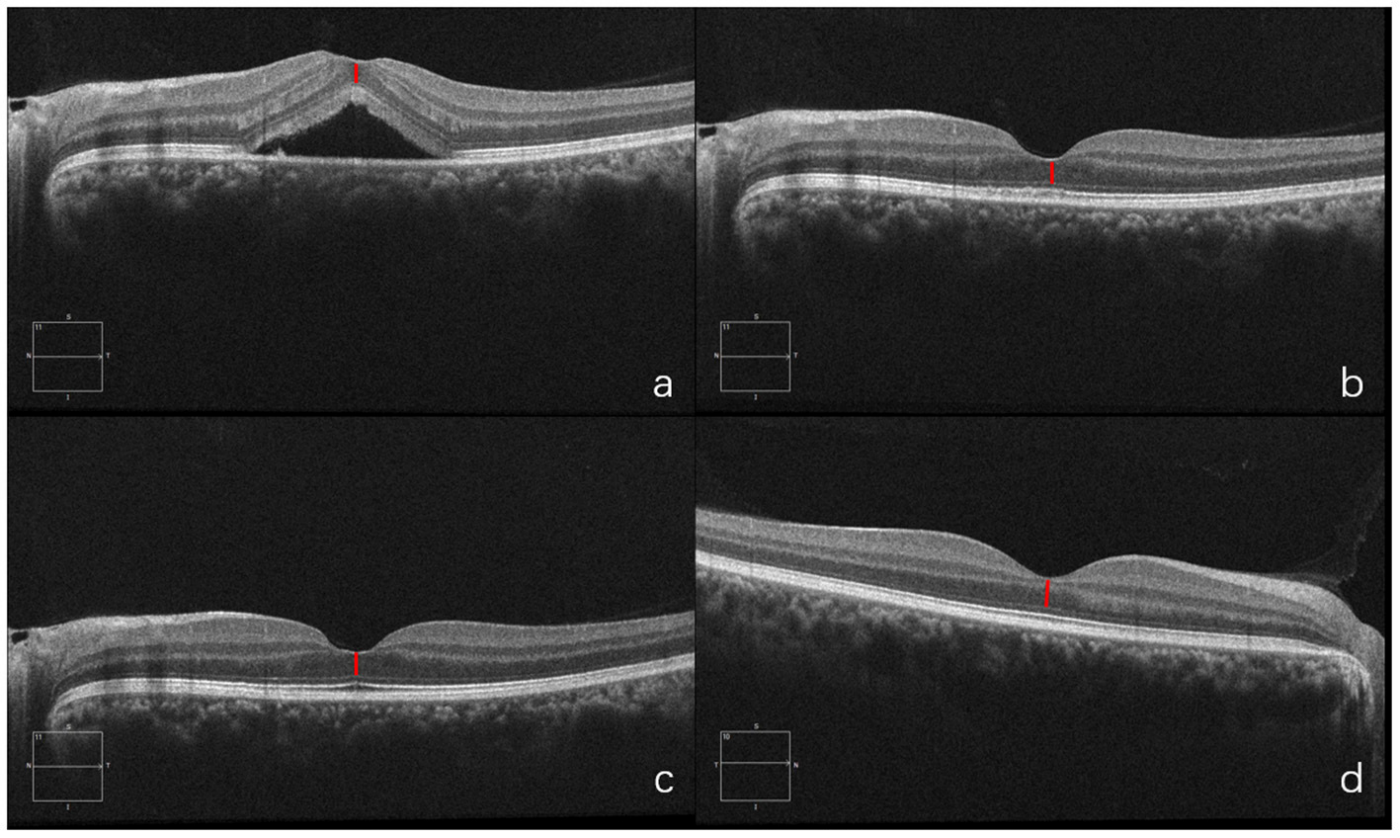
OCT of diseased eye before PDT **(a)**. 1 month after PDT **(b)**. 6 months after PDT **(c)** and of the fellow healthy eye **(d)**.

A 26-year-old man presented with a three-day history of blurred vision in the right eye. The physical examination showed that the BCVA in his right eye was 20/20. SD-OCT showed serous retinal detachment at the macula and the foveal ONL thickness was 102 μm. He was diagnosed with CSC in the right eye and chose to be treated with half-dose PDT immediately. One month after PDT, ONL thickness was 103 μm. At 6th month follow-up, fovea ONL thickness was 102 μm while his left eye showed fovea ONL thickness of 101 μm. This case illustrates that timely PDT treatment soon after onset could prevent damage to foveal ONL (Figure 7).

#### Case 2

**Figure 8:**
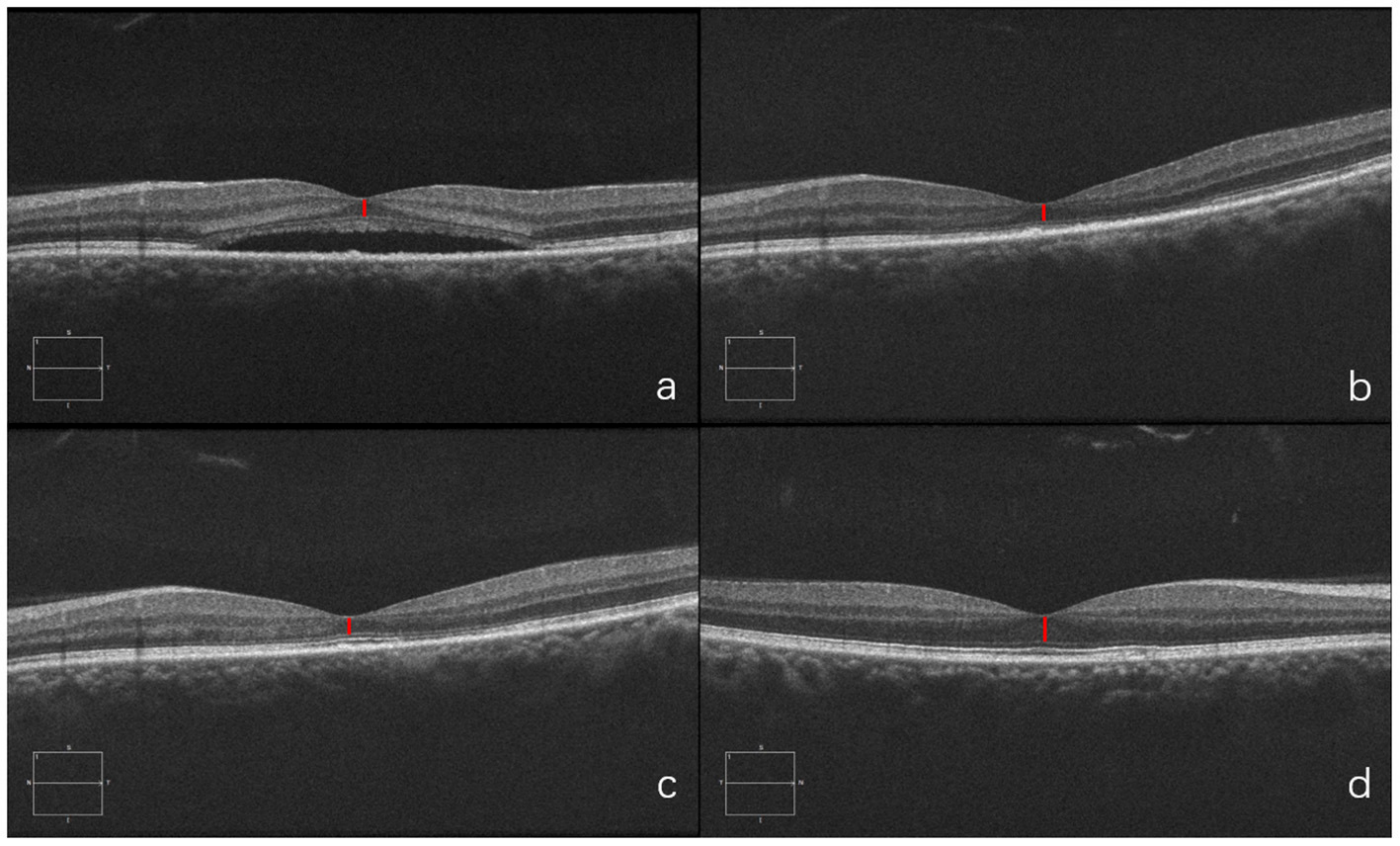
Optical coherence tomography (OCT) of the diseased eye before PDT **(a)**. 1 month after PDT **(b)**. 6 months after PDT **(c)**. and of the fellow healthy eye **(d)**.

A 34-year-old man presented with a five-month history of blurred vision in the left eye. The physical examination showed that the BCVA in his left eye was 20/50. SD-OCT showed serous retinal detachment at the macula and the foveal ONL thickness was 69 μm. He was treated with half-dose PDT. One month after PDT, foveal ONL thickness was 70 μm though the subretinal fluid had been absorbed. Six months later, the fovea ONL remained at 72 μm in the left eye while ONL of the right eye was 108 μm, indicating no significant recovery. This case demonstrates that in patients with longer disease duration, even after successful treatment, structural damage to the ONL remains irreversible (Figure 8).

#### Case 3

**Figure 9:**
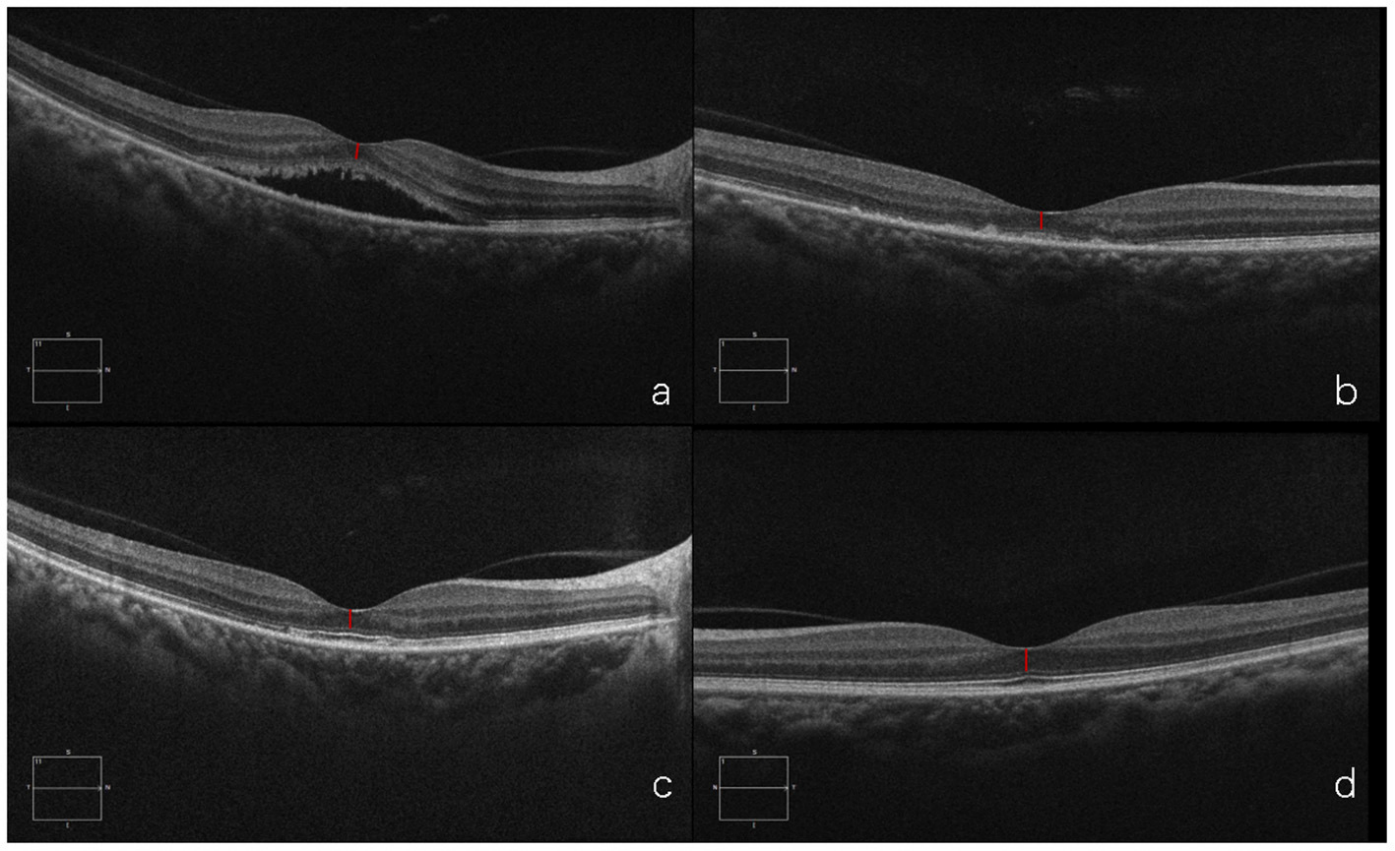
Optical coherence tomography (OCT) of the diseased eye before PDT **(a)**. 1 month after PDT **(b)**. 6 months after PDT **(c)**. and of the fellow healthy eye **(d)**.

A 37-year-old woman presented with a two-months history of blurred vision in the left eye. The physical examination showed that the BCVA in her left eye was 20/40. SD-OCT showed serous retinal detachment at the macula and the foveal ONL thickness was 79 μm. She was treated with half-dose PDT. One month after PDT, foveal ONL thickness was 81 μm as the subretinal fluid had been absorbed. Six months later, the foveal ONL regained to 101 μm in the left eye and ONL of the right eye was 110 μm, with an obvious recovery. This case demonstrates that in patients with duration>1 and ≤3 months, ONL thickness could still recover after PDT (Figure 9).

## Discussion

Our findings revealed that foveal ONL is thinner with longer pre-treatment duration of CSC. ONL thickness was substantially improved after treatment with PDT, but recovery was again related to disease duration prior to PDT.

The microstructure of the macular area in CSC patients, particularly the structure of the ONL, was essential with increasing concern. Previous investigations have documented considerably decreased ONL thickness in patients with visual loss (28, 29) with some claiming that CSC patients' vision loss was caused by photoreceptor apoptosis (30, 31). Similarly, our findings indicated gradual thinning of ONL with the progression of the disease, and with SRF persistence. Ozgur et al. found that photoreceptor cell layer atrophy occurs around 4 months after the onset of CSC symptoms (15), while Ozdemir et al. found that photoreceptor loss may begin within the first 3 months of CSC onset and that ONL thickness (photoreceptor as primary structure) was negatively correlated with symptom duration (16). Our findings indicate that foveal ONL thickness is a more sensitive indicator with variable degrees of ONL destruction happening as early as 1 week after the onset of the disease.

Foveal ONL thickness is not only associated with disease duration prior to therapy, it is also a powerful predictor of the patient's prognosis after treatment (19).In line with prior research, our findings showed that after PDT, ONL thickness gradually recovered along with CSC remission. The degree of recovery, however, differed between patients with different disease durations despite SRF having been completely absorbed after PDT. SRF-induced retinal stretch, according to Jia Yu et al., contributes significantly to the reduction in foveal ONL thickness (22). However, our findings imply that removal of SRF would not result in instant thickening of the ONL, and this is consistent with our previous research indicating that the effect of the subretinal fluid stretching can be ignored (data not shown). After SRF absorption, anatomical modification of the ONL structure appeared to be more relevant.

Fortunately, the ONL thickness of patients with a disease duration of less than 3 months recovered significantly at 6 months follow-up after PDT compared to pre-treatment. In addition, the ONL thickness in the affected eye was not substantially different from that in the healthy eye in individuals with a disease duration of less than 1 month. However, in those with disease duration over 3 months ONL thickness was not restored even after complete absorption of subretinal fluid, indicating that the ONL anatomy is not easily restored when SRF has been present for longer than 3 months. So far, there is no definite explanation to the recovery of ONL thickness after PDT for CSC patients. One possible explanation for restoration after shorter disease duration is that the ONL anatomy undergoes total remodeling if duration is less than 1 month. The ONL thickness showed partial remodeling after duration of 1 to 3 months, implying that the layer had been partially destroyed. Another explanation, according to Matsumoto et al., is that a recovery phagocytic cycle by RPE could be the cause of photoreceptor recovery in CSC. After PDT, the RPE may re-establish the photoreceptor phagocytic cycle, allowing the ONL to recover after the SRF is resolved (20). Even if SRF is absorbed, however, severe structural deterioration of the ONL occurs when symptoms have been present for 3 months. This indicates that if therapy is postponed, ONL damage may become permanent as the disease advances.

The question of whether CSC should be treated as soon as feasible is currently proving controversial. Previous studies have demonstrated that observation is the first-line treatment for patients with disease duration of less than 3 months (4). The neurosensory retina would be reattached in 3 months for most patients, according to Yannuzzi et al. (32). If the SRF has not been absorbed after 4 months, or if the other eye has had an incident with a poor visual prognosis, intervention is required (33). However, Loo et al. found that the persistence and recurrence of SRF could lead to vision loss below 20/40 in the long term (34). This is consistent with the present findings that persistence of SRF for long time result in poor vision and difficulty in recovery of ONL. While PDT is not frequently employed, clinicians may be obliged to observe the patient and alleviate any systemic factors that may impair CSC. Even though CSC is a self-limiting condition, about half of cases do not heal without treatment. Approximately 20–30% of patients will have one or more recurrence, with 5% developing chronic CSC, with permanent loss of vision (35). Previous investigations have found that the rate of anatomical response and VA improvement cloud to be almost 75 and 96% after PDT, respectively (10, 36, 37). PDT has been shown to be safe and effective in the treatment of CSC and our findings demonstrate that early PDT (within a month of the onset of the disease) can restore ONL to normal levels. According to our research, ONL is not thinned or significantly damaged within 1 week of disease onset, and patients treated promptly at this time have a better prognosis (see Typical Case 1). In summary, due to its effect on visual function and based on the facts presented above, we suggest that PDT should be started as soon as possible after CSC onset.

After PDT, all patients had some degree of visual acuity recovery, which was consistent with ONL thickness recovery. Interestingly, significant BCVA improvement was found in individuals with disease duration greater than 3 months, but not in those with duration less than 3 months. One possible explanation is that visual impairment was less severe in patients with recent onset disease. For patients with disease duration less than 3 months, not severe damage to BCVA has even occurred. As a result, the recovery of BCVA was not significant. Patients with duration shorter than 3 months, on the other hand, still had better final visual acuity than those with duration longer than 3 months. It was established that without spontaneous remission of the CSC, visual acuity will deteriorate over time. However, with PDT, vision can recover even if the condition lasts longer than 3 or 6 months. This also implies that, even if therapy is postponed, it is worthwhile.

This research has some limitations. Firstly, this is a retrospective study. Secondly, despite the sample size of 358 patients, we only looked at the foveal ONL and BCVA changes following PDT on 56 individuals. These were individuals with completely restored subretinal fluid and 6 months of follow up. Some patients declined to continue with the follow-up after the subretinal fluid was completely absorbed, while others underwent a second PDT and thus were removed from further participation. Thirdly, the follow up period was insufficient. Future research should include a larger sample followed up for a longer period to understand the long-term changes in foveal ONL after PDT.

## Conclusion

In summary, longer disease duration before treatment results in a thinner foveal ONL and lower likelihood of recovery after PDT. We suggest that CSC patients should be treated with PDT as soon as possible to prevent disease development and visual function loss.

## Data Availability Statement

The raw data supporting the conclusions of this article will be made available by the authors, without undue reservation.

## Ethics Statement

The studies involving human participants were reviewed and approved by Medical Ethics Committee of Peking University People's Hospital. The patients/participants provided their written informed consent to participate in this study. Written informed consent was obtained from the individual(s) for the publication of any potentially identifiable images or data included in this article.

## Author Contributions

MZ, YS, and XS: performing the screening and diagnosis of CSC. KD, YG, ZL, and YC: collection and assembly of data. KD and YG: data analysis and interpretation. All authors contributed to the study conception and design, manuscript writing, and final approval of manuscript.

## Funding

This work was supported by the National Natural Science Foundation of China Grant (82171060, 81970815); National key research and development program (2020YFC2008203); Beijing Residency Training Quality Improvement Project (No. Zhupei2021043).

## Conflict of Interest

The authors declare that the research was conducted in the absence of any commercial or financial relationships that could be construed as a potential conflict of interest.

## Publisher's Note

All claims expressed in this article are solely those of the authors and do not necessarily represent those of their affiliated organizations, or those of the publisher, the editors and the reviewers. Any product that may be evaluated in this article, or claim that may be made by its manufacturer, is not guaranteed or endorsed by the publisher.
