# Supplementary material for: Changes in the Foveal Outer Nuclear Layer of Central Serous Chorioretinopathy Patients Over the Disease Course and Their Response to Photodynamic Therapy
Source: Front Med (Lausanne). 2022 Jan 14;8:824239. doi: 10.3389/fmed.2021.824239 (PMC8795370; doi:10.3389/fmed.2021.824239)
Supplement: Supplementary file 1 [file Data_Sheet_1.PDF]

Table S1 Correlation between various factors with ONL thickness change.

| **Recovery** | **Factors** | **Mean/r** | **P** |
|---|---|---|---|
| | Gender(n) | mean±SD | P |
| Mean ONL change(μm) | Male(43) | 11.9±2.6 | 0.157 |
| | Female(13) | 20.1±5.6 | |
| **Spearman correlation** | | r | P |
| ONL thickness change(μm) | Age(year) | 0.159 | 0.241 |
| | Height of retinal detachment(μm) | -0.056 | 0.679 |
| | Width of subretinal space(μm) | 0.145 | 0.288 |
| | Ratio of width and height | 0.035 | 0.797 |
| | Best corrected visual acuity | 0.021 | 0.876 |

P>0.05 indicates that no significant difference of ONL thickness change.
